# Hospital-based preventative interventions for people experiencing homelessness in high-income countries: A systematic review

**DOI:** 10.1016/j.eclinm.2022.101657

**Published:** 2022-10-22

**Authors:** Serena A. Luchenski, Joanna Dawes, Robert W. Aldridge, Fiona Stevenson, Shema Tariq, Nigel Hewett, Andrew C. Hayward

**Affiliations:** a Collaborative Centre for Inclusion Health, Institute of Epidemiology and Healthcare, University College London, 1-19 Torrington Place, London WC1E 7HT, United Kingdom; b Centre for Public Health Data Science, Institute for Health Informatics, University College London, 255 Euston Road, London NW1 2DA, United Kingdom; c Department of Primary Care and Population Health, Institute of Epidemiology and Healthcare, University College London, Royal Free Hospital, Rowland Hill Street, London NW3 2PF, United Kingdom; d Centre for Clinical Research in Infection and Sexual Health, Institute for Global Health, University College London, Mortimer Market Centre, off Capper Street, London WC1E 6JB, United Kingdom; e Pathway, 4th Floor, East, 250 Euston Rd, London NW1 2PG, United Kingdom

**Keywords:** Homeless persons, Prevention, Hospital, Social determinants of health, Multicomponent interventions, Health inequalities, ED, Emergency Department, UK, United Kingdom, US, United States, RCT, Randomised Controlled Trial

## Abstract

**Background:**

People experiencing homelessness have significant unmet needs and high rates of unplanned care. We aimed to describe preventative interventions, defined in their broadest sense, for people experiencing homelessness in a hospital context. Secondary aims included mapping outcomes and assessing intervention effectiveness.

**Methods:**

We searched online databases (MEDLINE, Embase, PsycINFO, HMIC, CINAHL, Web of Science, Cochrane Library) from 1999–2019 and conducted backward and forward citation searches to 31 December 2020 (PROSPERO CRD42019154036). We included quantitative studies in emergency and inpatient settings measuring health or social outcomes for adults experiencing homelessness in high income countries. We assessed rigour using the “Quality Assessment Tool for Quantitative Studies” and summarised findings using descriptive quantitative methods, a binomial test, a Harvest Plot, and narrative synthesis. We used PRISMA and SWiM reporting guidelines.

**Findings:**

Twenty-eight studies identified eight intervention types: care coordination (*n=*18); advocacy, support, and outreach (*n=*13); social welfare assistance (*n=*13); discharge planning (*n=*12); homelessness identification (*n=*6); psychological therapy and treatment (*n=*6); infectious disease prevention (*n=*5); and screening, treatment, and referrals (*n=*5). The evidence strength was weak (*n=*16) to moderate (*n=*10), with two high quality randomised controlled trials. We identified six outcome categories with potential benefits observed for psychosocial outcomes, including housing (11/13 studies, 95%CI=54.6–98.1%, p=0.023), healthcare use (14/17, 56.6–96.2%, p=0.013), and healthcare costs (8/8, 63.1–100%, p=0.008). Benefits were less likely for health outcomes (4/5, 28.3–99.5%, p=0.375), integration with onward care (2/4, 6.8–93.2%, p=1.000), and feasibility/acceptability (5/6, 35.9–99.6%, p=0.219), but confidence intervals were very wide. We observed no harms. Most studies showing potential benefits were multi-component interventions.

**Interpretation:**

Hospital-based preventative interventions for people experiencing homelessness are potentially beneficial, but more rigorous research is needed. In the context of high needs and extreme inequities, policymakers and healthcare providers may consider implementing multi-component preventative interventions.

**Funding:**

SL is supported by an NIHR Clinical Doctoral Research Fellowship (ICA-CDRF-2016-02-042). JD is supported by an NIHR School of Public Health Research Pre-doctoral Fellowship (NU-004252). RWA is supported by a Wellcome Clinical Research Career Development Fellowship (206602).

**Research in context**

**Evidence before this study**

People experiencing homelessness have very high levels of unmet health and social needs. Hospital-based strategies are needed to ‘make every contact count’ with the health system. Evidence is lacking on how hospitals can help to improve outcomes and prevent future ill health and unplanned hospital care among people experiencing homelessness.

**Added value of this study**

This is the first systematic review to comprehensively describe hospital-based preventative interventions, defined in their broadest sense, and examine the effectiveness of these interventions for improving the wider health and social needs of people experiencing homelessness. We searched online databases (MEDLINE, Embase, PsycINFO, HMIC, CINAHL, Web of Science, Cochrane Library) from 1999-2019 and included quantitative studies of preventative interventions for adults experiencing homelessness living in high income countries. We found 40% of included studies were of moderate or high quality, most interventions included multiple components addressing both health and social needs, and outcomes for psychosocial factors (including housing), healthcare care utilisation, and healthcare costs improved most consistently.

**Implications of all the available evidence**

The evidence indicates there are important potential benefits of hospital-based preventative interventions in both inpatient and emergency hospital settings, but there is a relative scarcity of high-quality research. In the context of extreme health burden and inequity, action is urgently needed to improve outcomes for people experiencing homelessness. Policymakers and healthcare providers may wish to consider implementing and evaluating preventative interventions. Further high-quality research is needed for preventative interventions in a hospital context, particularly for screening for communicable and non-communicable diseases, infectious disease prevention, and how best to implement preventative interventions to ensure they are feasible and acceptable to staff and people experiencing homelessness.

Alt-text: Unlabelled box

## Introduction

People experiencing homelessness lack a safe, decent, and secure place to live, such as people sleeping on the street or living in temporary accommodation including hostels, squats, and insecure conditions with friends and family.^1^ The health of people experiencing homelessness may be understood as syndemic, whereby micro and macro-level factors, such as poverty, trauma, social exclusion, lack of affordable housing and limited access to healthcare, intersect and cause population-level clustering of diseases.^2^^,^^3^ Previous research has observed the common co-occurrence of and negative synergies between physical illnesses, mental illnesses, and substance use disorders in this population.^4^^,^^5^ Typically, people experiencing homelessness have two to five times higher incidence of mortality and morbidity across all diseases compared to the general population.^6^ In spite of their high health needs, people experiencing homelessness have poor access to primary and preventative care.^7^, ^8^, ^9^, ^10^ There are many barriers to access, such as stigma within services and society, competing priorities such as food, shelter, and addiction needs,^9^, ^10^, ^11^ and system-level barriers such as fragmented and inflexible services, poorly trained staff, and inadequate funding.^12^ Poor primary care access and high health needs are key drivers of unplanned hospital care utilisation among this population.^13^ Nearly a third of deaths among hospital patients experiencing homelessness in England were found to be due to causes amenable to timely and effective healthcare, relative to about a quarter of deaths amongst the most deprived housed population.^14^

System-wide action is needed to improve health and determinants of health for people experiencing homelessness. Hospital attendances are an opportunity to ‘make every contact count’^15^^,^^16^ and address this population's wider health and social needs through preventative interventions, in addition to the acute care response. Preventative interventions are broadly defined as, “policies and actions to eliminate a disease or minimise its effect; to reduce the incidence and/or prevalence of disease, disability, and premature death; to reduce the prevalence of disease precursors and risk factors in the population; and, if none of these is feasible, to retard the progress of incurable disease”.^17^ Preventative interventions are an essential part of healthcare systems,^18^, ^19^, ^20^, ^21^ but are often under-utilised.^19^ A significant proportion of the extreme burden of poor health faced by people experiencing homelessness is likely preventable with existing interventions,^22^ such as substance use treatment,^23^, ^24^, ^25^, ^26^ screening and treatment of infectious diseases,^7^^,^^27^, ^28^, ^29^ case management,^30^^,^^31^^,^^32^ intermediate care programmes for people experiencing homelessness leaving hospital,^30^ and ‘Housing First’.^30^^,^^33^ However, research is limited on the provision of preventative interventions for people experiencing homelessness in a hospital context.

Previous systematic reviews of interventions for people experiencing homelessness have been largely conducted in a community context,^30^^,^^22^^,^^34^, ^35^, ^36^, ^37^, ^38^, ^39^ with the exception of two recent reviews.^40^^,^^41^ Cornes et al.^40^ conducted a realist synthesis of hospital discharge interventions with a focus on intermediate care provision following an inpatient admission. Formosa et al.^41^ reviewed interventions for improving housing in the emergency department (ED). This is the first systematic review to take a comprehensive and holistic approach to examining preventative interventions in their broadest sense, from clinical interventions to action on social determinants of health, for people experiencing homelessness in a hospital setting. Our primary aim was to summarise the types of preventative interventions which have been evaluated for people experiencing homelessness in ED and inpatient settings. Secondary aims included mapping outcomes which have been used to measure benefits and to assess effectiveness of these interventions. PRISMA^42^ and SWiM^43^ reporting guidelines were used to describe the study.

## Methods

### Eligibility criteria

We used the ‘Population, Intervention, Comparison, Outcome’ (PICO) framework^44^ to define eligibility. We examined people experiencing homelessness over the age of 16 years (age of consent for medical treatment) living in high-income countries. Studies were included if they reported any homeless participants, whether disaggregated data were presented or not, because we aimed to identify all interventions which have the potential to benefit people experiencing homelessness, rather than seeking to report definitive outcome/effectiveness data. Any preventative interventions delivered in an acute hospital setting, including inpatient wards, mental health hospitals, and EDs, were eligible for inclusion. We did not have specific criteria relating to comparison groups or outcomes. Quantitative research studies (experimental, quasi-experimental, and observational) published in English between 1999 and 2019 were included. We did not include protocols, unpublished manuscripts, or conference abstracts. We excluded reviews from this study but selected the underlying studies meeting inclusion criteria.

### Information Sources and Search Strategy

We conducted searches on 8 October 2019 in the following electronic databases: MEDLINE via Ovid; Embase via Ovid; PsycINFO via Ovid; HMIC via Ovid; CINAHL via EBSCOhost; Web of Science; and The Cochrane Library. Searches were limited to English language studies published between 1999-2019. The search strategy included keywords and subject headings for homelessness AND hospitals AND preventative interventions. We developed a comprehensive list of potential preventative interventions relevant to people experiencing homelessness from an initial scoping review and discussion between expert homeless health clinicians to inform our search strategy. This preliminary work comprised of scientific literature searches within Medline as well as grey-literature searches in Google for preventative interventions for people experiencing homelessness and comprehensive prevention frameworks for the general population. SL (public health specialist) developed a list of potentially relevant preventative interventions from these sources and categorised them by International Classification of Disease (ICD-10) chapter. SL shared this list with expert clinicians working in hospital and community settings with people experiencing homelessness, including a general practitioner (co-author NH), a senior nurse (SDS, acknowledgement), and two public health doctors (co-authors RA and AH). We sought feedback about anything missing or irrelevant to people experiencing homelessness and adapted the list with the aim of being as inclusive as possible. Using the finalised list of potential preventative interventions (see Appendix 1), we developed a set of keyword and subject search terms for preventative interventions. Detailed search terms for each database can be found in Appendix 2. We also hand searched reference lists of any relevant systematic reviews identified in database searches and reference lists of included papers (backward citation search). We ran an automated forward citation search of included papers using Web of Science on 31 December 2020.

### Study selection and data collection

One reviewer (SL) screened titles and abstracts of studies retrieved from database searches and conducted backward and forward citation searches. Two reviewers (SL and JD) independently assessed full texts against predefined criteria (Kappa = 0.79). Discrepancies were resolved through discussion. Endnote referencing software was used to import references, screen titles and abstracts, and to read and select full text articles. One reviewer (SL) extracted data from selected studies, and a second reviewer (JD) independently checked all extracted data. Again, discrepancies were resolved through discussion. Data were extracted using a piloted, standardised Google Sheet (online spreadsheet) for: first author, year, title, research aim, study design, country, hospital setting, recruitment and follow-up, study population description, proportion experiencing homelessness, eligibility criteria, intervention characteristics, multicomponent/ stand-alone intervention, comparisons, sample size, outcomes, direction of effects, and measures of effectiveness. All outcomes and measures of effectiveness were collected and described as reported in the original research articles. A unique identifier was given to individual studies to aid cross-referencing between tables, figures, and appendices.

### Quality assessment

We used the “Quality Assessment Tool for Quantitative Studies” to assess rigor.^45^^,^^46^ This tool was selected because it has been developed and validated specifically for public health interventions and can be applied to articles of any public health topic area, including prevention (available from: https://www.ephpp.ca/quality-assessment-tool-for-quantitative-studies/). The tool includes an assessment (with a rating of strong, moderate, or weak) of the following characteristics: selection bias, study design, confounders, blinding, data collection methods, withdrawals and dropouts, intervention integrity, and analysis. A global rating is then given to each paper where strong corresponds to no weak ratings, moderate to one weak rating, and weak to two or more weak ratings. One reviewer (SL) completed the quality assessment tool, and another (JD) checked the ratings. We resolved disagreements through discussion.

### Synthesis methods

We summarised studies using descriptive quantitative methods, visual displays, and narrative synthesis using guidance from Chapter 12 of the Cochrane Handbook for Systematic Reviews of Interventions.^47^ Based on an initial scoping review we determined it was not possible to do a meta-analysis because of the lack of standardised outcome data.^47^ We developed categories of preventative interventions and outcomes from included studies to summarise findings and assigned studies to as many categories as relevant. Quantitative analysis involved vote counting of key study characteristics and the number of effective interventions by intervention and outcome category. Vote counting is the recommended synthesis method when there is a lack of consistent effect measures across studies.^47^ It aims to compare the number of effects with evidence of potential benefit to the number of effects showing no benefit for a particular outcome using solely the direction of effect. Conventional methods using statistical significance, magnitude of effect, or subjective rules such as study size cut-offs have been shown to be problematic.^47^ We produced a standardised binary metric (benefit or no benefit based on direction of effect) which we used to calculate a proportion, 95% confidence interval (binomial exact calculation), and p-value (binomial probability test) to demonstrate if there is any evidence of an effect^47^ for preventative interventions overall, by outcome category. When categorising effects as beneficial or not where there were multiple outcomes within an outcome category (e.g. multiple healthcare utilisation outcomes), we categorised the intervention as having evidence of benefit if *any* of the outcomes favoured the intervention because the main purpose of this review was to identify interventions with the *potential* to benefit people experiencing homelessness in hospital. We then produced a summary table of effectiveness and a Harvest Plot^47^^,^^48^ which is a ‘supermatrix’ visually displaying the results from the vote counting for each intervention and outcome combination. We also produced a textual summary synthesising findings and evidence quality according to intervention and outcome categories.

### Role of funding source, registration, and protocol

The funders of the study had no role in study design, data collection, data analysis, data interpretation, or writing of the report. Only the research team had access to the raw data. The protocol for this review was registered on the Prospero database (CRD42019154036) on 28 October, 2019.^49^

## Results

**Figure 1:**
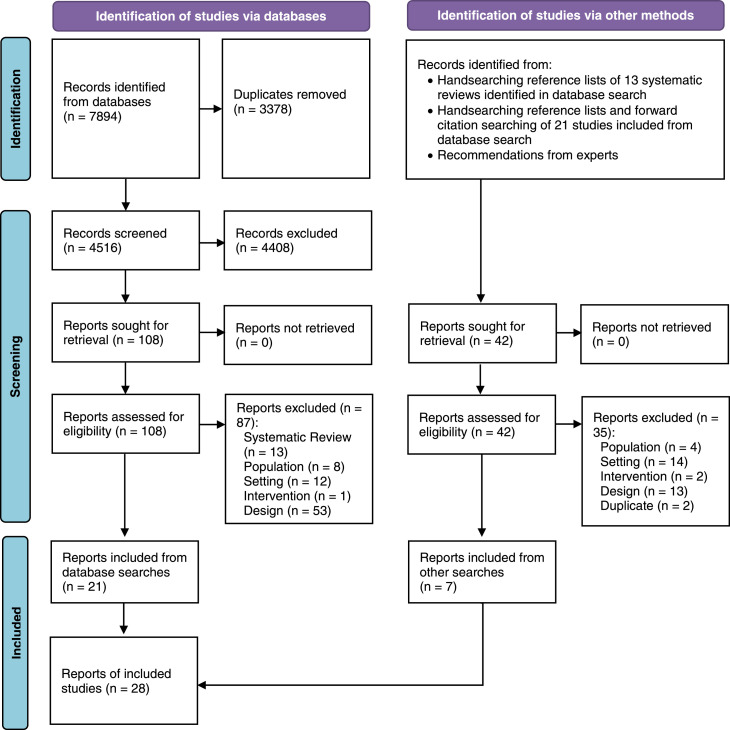
**Selection of Included Studies - PRISMA Flow Diagram**. A record refers to an entry in an electronic database describing a report. A report is a full text published research article and there may be one or more reports describing an individual research study. In this review, each report described a unique research study (*n=* 28 reports = 28 unique research studies).

We identified 7,894 records from electronic database searches and screened 4,516 records after duplicates were removed. We reviewed 108 full-text reports, of which 21 were included. Handsearching identified an additional seven reports. We included a total of 28 reports, corresponding to 28 unique research studies. Full details of the selection process and reasons for exclusion are provided in Figure 1.

Studies were published in the US (*n=*13),^50^, ^51^, ^52^, ^53^, ^54^, ^55^, ^56^, ^57^, ^58^, ^59^, ^60^, ^61^ UK (*n=*8),^62^, ^63^, ^64^, ^65^, ^66^, ^67^, ^68^, ^69^ Australia (*n=*4),^70^, ^71^, ^72^, ^73^ and Canada (*n=*3)^74^, ^75^, ^76^ and were similarly distributed across inpatient (*n=*12)^59^^,^^60^^,^^62^^,^^64^, ^65^, ^66^, ^67^, ^68^, ^69^^,^^73^^,^^74^^,^^76^ and emergency settings (*n=*15),^50^, ^51^, ^52^, ^53^^,^^55^, ^56^, ^57^, ^58^^,^^61^^,^^63^^,^^70^, ^71^, ^72^^,^^75^ with one study conducted in both inpatient and ED settings.^54^ Half were conducted in exclusively homeless populations (*n=*14),^50^^,^^51^^,^^54^^,^^55^^,^^60^^,^^62^^,^^64^, ^65^, ^66^, ^67^, ^68^, ^69^^,^^73^^,^^76^ while the others had a mix of homeless and other patient groups (*n=*14).^52^^,^^53^^,^^56^, ^57^, ^58^, ^59^^,^^61^^,^^63^^,^^70^, ^71^, ^72^^,^^74^^,^^75^ Over half of studies were classified as methodologically weak (*n=*16).^51^^,^^53^^,^^54^^,^^58^^,^^62^^,^^63^^,^^66^, ^67^, ^68^, ^69^, ^70^, ^71^, ^72^, ^73^, ^74^, ^75^ There were five randomised controlled trials,^56^^,^^60^^,^^61^^,^^65^^,^^76^ two of which were classified as high quality.^60^^,^^65^ Counts and proportions of key study characteristics are presented in Table 1.

**Table 1:** Characteristics of Included Studies, *N* = 28. Proportions do not total 100% if studies appear in more than one category. RCT = randomised controlled trial. Full study details are provided in Appendix 3.

Characteristics	n (%)	References
**Country**		
UK	8 (28.6)	62, 63, 64, 65, 66, 67, 68, 69
USA	13 (46.4)	50, 51, 52, 53, 54, 55, 56, 57, 58, 59, 60, 61
Australia	4 (14.3)	70, 71, 72, 73
Canada	3 (10.7)	74, 75, 76
**Study Design**		
RCT	5 (17.9)	^56^^,^^60^^,^^61^^,^^65^^,^^76^
Non-Randomised Trial	1 (3.6)	^55^
Cohort (including one group and		
two group before/after studies)	15 (53.6)	^50^^,^^52^^,^^57^, ^58^, ^59^^,^^64^^,^^67^, ^68^, ^69^^,^^71^, ^72^, ^73^, ^74^, ^75^
Time Series	3 (10.7)	^53^^,^^54^^,^^70^
Cross-Sectional	4 (14.3)	^51^^,^^62^^,^^63^^,^^66^
**Hospital Department**		
Inpatients Exclusively	12 (42.9)	^59^^,^^60^^,^^62^^,^^64^, ^65^, ^66^, ^67^, ^68^, ^69^^,^^73^^,^^74^^,^^76^
Emergency Exclusively	15 (53.6)	50, 51, 52, 53^,^^55^, ^56^, ^57^, ^58^^,^^61^^,^^63^^,^^70^, ^71^, ^72^^,^^75^
Both Inpatient and Emergency	1 (3.6)	^54^
**Study Population**		
Exclusively homeless	14 (50.0)	^50^^,^^51^^,^^54^^,^^55^^,^^60^^,^^62^^,^^64^, ^65^, ^66^, ^67^, ^68^, ^69^^,^^73^^,^^76^
Mixed homeless and other	14 (50.0)	^52^^,^^53^^,^^56^, ^57^, ^58^, ^59^^,^^61^^,^^63^^,^^70^, ^71^, ^72^^,^^74^^,^^75^
**Preventative Intervention Category**		
Care Coordination	18 (64.3)	^51^^,^^52^^,^^55^^,^^57^, ^58^, ^59^, ^60^, ^61^, ^62^^,^^64^, ^65^, ^66^, ^67^^,^^69^^,^^71^, ^72^, ^73^^,^^75^
Advocacy, Support, & Outreach	13 (46.4)	^55^^,^^57^, ^58^, ^59^^,^^61^^,^^64^, ^65^, ^66^, ^67^^,^^69^^,^^71^^,^^73^^,^^76^
Social Welfare Assistance	13 (46.4)	^52^^,^^55^^,^^57^, ^58^, ^59^, ^60^, ^61^, ^62^^,^^66^^,^^67^^,^^69^^,^^71^^,^^76^
Discharge Planning	12 (42.9)	^55^^,^^59^^,^^60^^,^^62^^,^^64^, ^65^, ^66^, ^67^, ^68^, ^69^^,^^73^^,^^76^
Homelessness Identification	5 (17.9)	^50^^,^^51^^,^^71^^,^^73^^,^^75^
Psychological Therapy/Treatment	6 (21.4)	^56^^,^^58^^,^^61^^,^^74^^,^^75^
Infectious Disease Prevention	5 (17.9)	^53^^,^^54^^,^^63^^,^^70^^,^^75^
Screening, Treatment, & Referral	5 (17.9)	^56^^,^^63^^,^^70^^,^^75^^,^^77^
**Outcome Categories**		
Health	6 (21.4)	^54^^,^^61^^,^^65^^,^^70^^,^^71^^,^^74^
Psychosocial	12 (46.4)	^55^^,^^56^^,^^58^^,^^61^^,^^62^^,^^64^, ^65^, ^66^^,^^68^^,^^71^^,^^72^^,^^76^
Integration	4 (14.3)	^58^^,^^63^^,^^70^^,^^72^
Healthcare Use	17 (60.7)	^50^^,^^52^, ^53^, ^54^, ^55^, ^56^, ^57^, ^58^, ^59^, ^60^, ^61^^,^^64^^,^^65^^,^^69^^,^^71^^,^^73^^,^^74^
Healthcare Costs	9 (32.1)	^52^^,^^58^^,^^59^^,^^61^^,^^65^^,^^67^^,^^71^, ^72^, ^73^
Feasibility and Acceptability	5 (17.9)	^51^^,^^53^^,^^63^^,^^70^^,^^75^
**Quality Rating**		
Strong	2 (7.1)	^60^^,^^65^
Moderate	10 (35.7)	^50^^,^^52^^,^^55^, ^56^, ^57^^,^^59^^,^^61^^,^^64^^,^^76^
Weak	16 (57.1)	^51^^,^^53^^,^^54^^,^^58^^,^^62^^,^^63^^,^^66^, ^67^, ^68^, ^69^, ^70^, ^71^, ^72^, ^73^, ^74^, ^75^

We identified eight preventative intervention categories: care coordination (*n=*18 studies);^51^^,^^52^^,^^55^^,^^57^, ^58^, ^59^, ^60^, ^61^, ^62^^,^^64^, ^65^, ^66^, ^67^^,^^69^^,^^71^, ^72^, ^73^^,^^75^ advocacy, support, and outreach (*n=*13);^55^^,^^57^, ^58^, ^59^^,^^61^^,^^64^, ^65^, ^66^, ^67^^,^^69^^,^^71^^,^^73^^,^^76^ social welfare assistance (*n=*13);^52^^,^^55^^,^^57^, ^58^, ^59^, ^60^, ^61^, ^62^^,^^66^^,^^67^^,^^69^^,^^71^^,^^76^ discharge planning (*n=*12);^55^^,^^59^^,^^60^^,^^62^^,^^64^, ^65^, ^66^, ^67^, ^68^, ^69^^,^^73^^,^^76^ homelessness identification (*n=*6);^50^^,^^51^^,^^71^^,^^73^^,^^75^ psychological therapy and treatment (*n=*6);^56^^,^^58^^,^^61^^,^^74^^,^^75^ infectious disease prevention (*n=*5);^53^^,^^54^^,^^63^^,^^70^^,^^75^ and screening, treatment, and referral (*n=*5).^56^^,^^63^^,^^70^^,^^75^^,^^77^ Most studies were multicomponent interventions and were classified in as many categories as relevant. Operational definitions of categories and examples of interventions are presented in Table 2. We found six outcome categories: health;^54^^,^^61^^,^^65^^,^^70^^,^^71^^,^^74^ psychosocial, including housing;^55^^,^^56^^,^^58^^,^^61^^,^^62^^,^^64^, ^65^, ^66^^,^^68^^,^^71^^,^^72^^,^^76^ integration with onward care outside the hospital;^58^^,^^63^^,^^70^^,^^72^ healthcare use;^50^^,^^52^, ^53^, ^54^, ^55^, ^56^, ^57^, ^58^, ^59^, ^60^, ^61^^,^^64^^,^^65^^,^^69^^,^^71^^,^^73^^,^^74^ healthcare costs and cost-effectiveness;^52^^,^^58^^,^^59^^,^^61^^,^^65^^,^^67^^,^^71^, ^72^, ^73^ and feasibility, acceptability, and engagement.^51^^,^^53^^,^^63^^,^^70^^,^^75^ Definitions and examples of outcome categories are presented in Table 3. Psychosocial, healthcare utilisation, and healthcare costs and cost-effectiveness were the most investigated outcomes across studies. Full study details and quality assessments are presented in Appendix 3 and 4, respectively.

**Table 2:** Operational definitions and examples of intervention categories.

Intervention Category	Operational Definition	Examples from Included Studies
Care Coordination	Coordination and integration of hospital teams with other services, such as primary care, intermediate care, housing, drug and alcohol, mental health, and others, to provide a holistic, person-centred package of care.	Case management; referrals and liaison between hospital and other services; multidisciplinary needs assessment and care planning; primary care in-reach; critical time intervention
Advocacy, Support, and Outreach	Support and advocacy provided by a health or social care professional, case manager, or peer, to assist with wider health and social needs.	Clinical or peer advocacy; support to book/attend appointments or complete forms; enhancing engagement and participation; enhancing motivation; support; persistent/assertive outreach
Social Welfare Assistance	Assistance with social and welfare needs.	Housing support; legal support; transport to appointments or accommodation; income assistance; applying for benefits or health insurance; necessities such as foodbank vouchers, clothing, toiletries, mobile phones
Discharge Planning	Planning for patients’ care needs after a stay in hospital, including appropriate housing and/or intermediate care.	Arranging suitable accommodation based on patients’ needs post-hospitalisation, such as supported housing or intermediate care
Homelessness Identification	Assessing if a person is experiencing homelessness to tailor care.	Screening questionnaire; alerts on electronic health records; clinical assessment
Psychological Therapy and Treatment	Psychosocial or pharmacological therapies/treatment for mental health and/or substance use.	Counselling; substance use treatment; individual/group therapy; crisis management; medications; brief intervention
Infectious Disease Prevention	Interventions designed to prevent contraction and spread of infectious diseases.	Vaccinations; hygiene kits; education/leaflets on handwashing and infectious disease prevention; hepatitis C treatment (treatment as prevention)
Screening, Treatment, and Referral	Detection of disease or risk factor to treat and/or refer for onward care.	Blood tests for hepatitis B/C; PAP smears; questionnaires and brief intervention for smoking, drug, or alcohol use

**Table 3:** Definitions and examples of outcome categories.

Outcome Category	Operational Definition	Examples from Included Studies	Potential Benefit	No Benefit
Health	Physical and/or mental health status	Quality of life; sustained viral response	Health improves	No impact or harmful impact
Psychosocial	Psychological or social factors	Substance use; housing; reconnection with family	Psychological or social factors improved	No impact or harmful impact
Integration	Intervention links people to care outside hospital	GP registration; follow-up care	People better linked in with services	No impact or harmful impact
Healthcare Use	Attendances at hospital or other healthcare service	ED presentations; hospital admissions; length of stay; outpatients visits	Reductions in unplanned care and/or increases in planned care	No impact or harmful impact
Healthcare Costs	Cost-effectiveness of intervention; healthcare costs	Cost-effectiveness ratio; total hospital care costs	Cost-effective at a defined level; reductions in healthcare costs	No impact or harmful impact
Feasibility and Acceptability	Ease of implementation and willingness to engage	Uptake rate; clinician's views of intervention; adherence to intervention	Good engagement by people experiencing homelessness and/or clinicians	No impact or harmful impact

**Figure 2:**
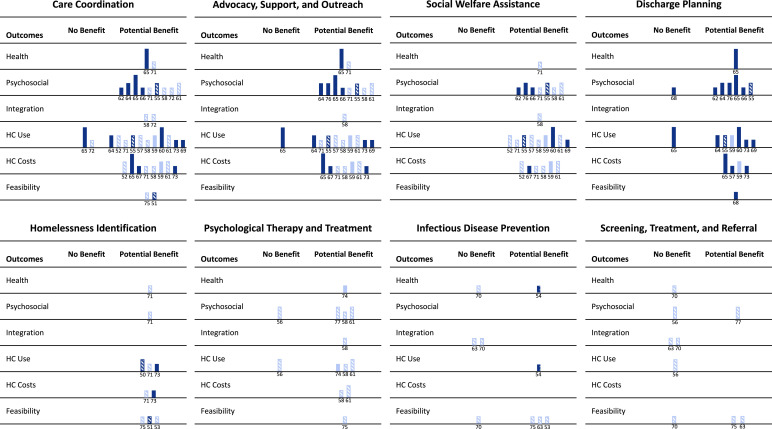
**Evidence for effectiveness of hospital-based preventative interventions for people experiencing homelessness by outcome category**. This Harvest Plot is a ‘supermatrix’ covering eight categories of interventions and six categories of outcomes. Each bar represents one study and is annotated with the reference number. Bar colour indicates population with dark blue depicting exclusively homeless populations and light blue showing mixed homeless and other populations. Bar height indicates study quality rating: strong (highest), moderate (medium), weak (lowest). Bar pattern indicates setting with stripes representing emergency departments exclusively, solid bars representing inpatients exclusively, and spotted bars representing both emergency and inpatient settings. *Notes: HC = Healthcare; Feasibility = Feasibility, Acceptability and Engagement.

The Harvest Plot (Figure 2) summarises findings for effectiveness (potential benefit versus no benefit) across intervention categories and outcomes, as well as presenting key descriptive characteristics for each study. The four intervention categories with most evidence (care coordination; advocacy, support, and outreach; social welfare assistance; and discharge planning) are displayed on the top row of the figure. Most studies in these categories showed beneficial effects for psychosocial, healthcare utilisation, and healthcare cost/cost-effectiveness outcomes from a range of strong, moderate, and weak quality studies. The studies included both mixed and exclusively homeless populations in both inpatient and ED settings. Results for integration appeared beneficial for the two weak-rated studies^58^^,^^72^ which measured this outcome across these four intervention categories. Health outcomes were measured by two studies,^61^^,^^65^^,^^71^ both of which identified potential benefits, including one strong RCT.^65^ Feasibility, acceptability, and engagement outcomes for care coordination and discharge planning interventions were measured by three weak-rated studies^51^^,^^68^^,^^75^ and findings showed evidence of potential benefit.

As can be seen from the overlap of study IDs from these four intervention categories with the most evidence (20 unique studies across 107 data points in the Harvest Plot), many studies included two or more interventions. The two high quality RCTs^60^^,^^65^ were both comprehensive multicomponent interventions involving multidisciplinary care coordination and discharge planning (as well as advocacy^65^ and social welfare assistance^60^) for people experiencing homelessness admitted to hospital. Hewett and colleagues^65^ showed the intervention was cost-effective, and it reduced homelessness. It indicated improvements in quality of life, but there were no effects on healthcare utilisation outcomes compared to usual care participants.^65^ In contrast, Sadowski et al.^60^ measured healthcare utilisation and demonstrated large reductions in the number of admissions, hospital bed days, and ED visits compared to the control group.

In the bottom row of the Harvest Plot, we display the four intervention categories with least evidence (homelessness identification; psychological therapy and treatment; infectious disease prevention; and screening, treatment, and referral). Four weak-rated studies^51^^,^^71^^,^^73^^,^^75^ and one moderate quality study^50^ examined homelessness identification as an intervention; results showed benefits for health, psychosocial, healthcare use, healthcare costs/cost-effectiveness, and feasibility/acceptability/engagement outcomes in both ED and inpatient settings. Integration outcomes were not measured. Psychological therapy and treatment interventions were assessed by three moderate-rated^56^^,^^61^^,^^77^ and three weak-rated^58^^,^^74^^,^^75^ studies. All except one study of a brief intervention for drug use^56^ showed benefits across all outcome categories, though none of these studies were conducted in an exclusively homeless population. Infectious disease prevention was investigated by five weak studies,^53^^,^^54^^,^^63^^,^^70^^,^^75^ which showed mixed findings in ED and inpatients settings and in exclusively homeless and mixed patient populations. Two of these studies,^63^^,^^70^ which were also categorised as screening, treatment, and referral interventions, showed no benefit for health and integration outcomes for hepatitis C. There were poorer rates of linkage to care, treatment uptake, and adherence, and failure to clear hepatitis C virus for people experiencing homelessness compared to housed populations following a positive screening test for hepatitis C. Two studies examined screening, brief intervention, and referral to treatment (SBIRT) interventions^56^^,^^77^ in an ED setting; both were of moderate quality. One study found the intervention reduced harmful alcohol behaviours,^77^ whilst the other found no reduction in drug use behaviours and no increase in uptake of drug treatment services.^56^ Three weak-rated studies^53^^,^^63^^,^^75^ demonstrated implementing infectious disease prevention and screening, treatment, and referral programmes in the ED was both feasible and acceptable.

We present a global summary of the effectiveness of preventative interventions by outcome category in Table 4. Similar to the Harvest plot, this table shows overall, hospital-based preventative interventions identified in this review are potentially beneficial for improving psychosocial, healthcare use and healthcare costs/cost-effectiveness outcomes, but less likely to be beneficial for health outcomes, integration with onward care, or feasibility/acceptability outcomes (p-value and 95% confidence interval are from the binomial probability test). No studies reported any intervention-associated harms.

**Table 4:** Overall effectiveness of preventative interventions by outcome category. **The *p*-value is produced from the binomial probability test, testing if the true proportion of effects favouring the intervention is equal to 0.5 (i.e. have occurred by chance).**

Outcome	No. Studies with Potential Benefit/Total No. Studies	Proportion of Beneficial Studies (%)	95% CI of the Proportion of Potentially Beneficial Studies	*p*-value
Health	4/5	80.0	(28.3 – 99.5)	0.375
Psychosocial	11/13	92.3	(54.6 – 98.1)	0.023
Integration	2/4	50.0	(6.8 – 93.2)	1.000
Healthcare Use	14/17	88.2	(56.6 – 96.2)	0.013
Healthcare Costs & Cost-Effectiveness	8/8	100.0	(63.1 – 100.0)	0.008
Feasibility, Acceptability & Engagement	5/6	83.3	(35.9 – 99.6)	0.219

## Discussion

This review highlights the range of preventative interventions which have been investigated for people experiencing homelessness in hospital settings. Overall, these interventions were shown to have potential benefits for psychosocial, healthcare use, and healthcare cost/cost-effectiveness outcomes. Four intervention categories (care coordination; advocacy, support, and outreach; social welfare assistance; and discharge planning) had the strongest evidence of benefit, including evidence from two high-quality RCTs. Most were multi-component complex interventions that sought to improve health and social needs in a person-centred way. They aimed to coordinate care with multiple agencies, plan for appropriate accommodation and care post-discharge, advocate for and support patients, and address wider health and social determinants of health of people experiencing homelessness. The strength of evidence was mixed with few high quality RCTs available on this topic, but two strong RCTs demonstrated the value of a multi-component preventative approach. Homelessness identification is an important intervention showing potential benefits, but with relatively limited evidence. Psychological therapy and treatment also had few studies showing evidence of beneficial effects. Infectious disease prevention and screening, treatment, and referral for communicable and non-communicable diseases are important preventative interventions, however there was a lack of evidence for how to effectively deliver these interventions in the hospital context.

Previous systematic reviews have also demonstrated the potential for hospital-based interventions to improve housing (psychosocial) outcomes for people experiencing homelessness.^40^^,^^41^ To our knowledge, this is the first time a review has demonstrated benefits of hospital-based preventative interventions for reducing unplanned healthcare utilisation and costs, highlighting the system-level benefits of a preventative approach for people experiencing homelessness to public hospital services which are already under pressure. We uncovered limited evidence for how to improve health directly and effectively link people into care following attendance at hospital. This was in part because health and integration outcomes were infrequently examined, similar to findings from reviews of case management^78^^,^^79^ and supportive housing^79^^,^^80^ in other settings. It was also because there were few studies of interventions which may target health outcomes more specifically, such as screening, treatment, and referral for communicable and non-communicable diseases. In contrast, reviews of community-based interventions have measured specific health outcomes such as mental health,^30^ diabetes,^35^ and liver-disease.^37^ This difference is likely related to the acute, short-term nature of hospital care compared to community care which is more focussed on long-term conditions.

The main strengths of our systematic review include the broad and inclusive nature of the review topic, comprehensive search strategy in electronic databases, and use of two reviewers for assessing full-text articles and data extraction. Another key strength is our use of vote counting, a sign test, and a Harvest Plot to synthesise intervention effectiveness in lieu of a meta-analysis and Forest Plot. Limitations, discussed below, relate to inclusion criteria, synthesis methods, and quality of the underlying studies.

Our inclusion criteria were not defined for a definitive review of intervention effectiveness in a specifically defined homeless-exclusive population. We included mixed populations because we aimed to identify the broadest range of potentially beneficial interventions and defining this population is notoriously challenging. Homelessness takes many forms from temporary or insecure accommodation to sleeping on the streets, with people often cycling in and out of homelessness.^1^ Furthermore, homelessness is not routinely and consistently recorded in hospital data systems^50^^,^^52^^,^^57^, ^58^, ^59^^,^^64^^,^^67^, ^68^, ^69^^,^^71^, ^72^, ^73^, ^74^, ^75^ and this results in underestimates of the true proportion of people experiencing homelessness attending hospital services. This latter issue may have also resulted in us missing studies of relevant interventions because housing status was not assessed or reported. We may have also missed relevant studies because of our chosen timeframe. On balance, we felt updating the review period to beyond 2019 would mean inclusion of studies conducted during the COVID-19 pandemic. Studies of preventative interventions conducted during this distinct period, such as COVID vaccinations, may not be generalisable in a non-pandemic context and would be better addressed in a separate review.

A limitation of the synthesis methods was we presented intervention components as separate categories for simplicity, but most interventions had multiple components and should be interpreted in the context of a wider package of care. It is not possible to know which components were most important, nor how they interacted with one another, because of lack of data. When multiple outcomes within an outcome category were measured, we categorised the intervention as having evidence of benefit if any outcomes favoured the intervention to identify all potentially beneficial interventions. This may have increased the likelihood of erroneously identifying benefit in studies that investigate large numbers of outcomes within a single category, without statistical correction for multiple comparisons. However, most included studies had three or less outcomes per category, and outcomes tended to all point in the same direction of benefit or no benefit (Appendix 3). We did not extract demographic data for people experiencing homelessness from included studies because of inconsistent reporting. This would have enabled us to take a more intersectional approach to understanding potentially important differences in intervention effectiveness between men and women, for different ethnicities, and age groups.

With respect to limitations of the underlying studies, there is possible publication bias given relatively few interventions showed no benefits. Inclusion of grey literature, conference proceedings, and protocols may have reduced this bias. Findings might also be biased by weak studies (57% of included studies) which observed intervention benefits when there are actually no true benefits (i.e. a type 1 error). The assessment tool we used may have scored studies higher than other widely used review tools.^46^ Importantly, the tool we used categorised before/after studies as ‘moderate’ and observed benefits may have resulted from regression to the mean. The two high-quality RCTs, however, were less likely to have these limitations and they supported the main review findings.

Further research is needed for preventative interventions that can directly improve health outcomes, such as psychological therapy and treatment, infectious disease prevention, and screening, treatment, and referral for non-communicable diseases, such as cancer, liver disease, and cardiovascular disease. In Appendix 1 (the list of theoretically important preventative interventions for people experiencing homelessness which we developed from a scoping review and expert opinion to inform the search strategy), we have highlighted which chapters of the International Classification of Disease (ICD-10) we identified interventions for and which we did not find any evidence for. This review uncovered only interventions related to three chapters: infectious diseases; mental illness and behavioural disorders (including drug and alcohol use disorders); and factors influencing health status and contact with health services. Without preventative interventions to improve leading causes of preventable death and ill health, such as cardiovascular disease, cancer, and tobacco use, there is a missed opportunity to intervene directly on health and reduce extreme inequities in morbidity and mortality of people experiencing homelessness. It is possible such work is being done in many hospitals internationally, however, more published evidence is needed to spread good practice and improve provision of preventative interventions in hospitals for people experiencing homelessness. Another research gap is how to implement and scale up preventative interventions in hospitals, including barriers and facilitators from the perspectives of key stakeholders and people experiencing homelessness. There was limited evidence for the feasibility and acceptability of these interventions in this review. Qualitative literature was beyond the scope of this review, but it may have provided contextual understanding of how various interventions work (or not) and why, as well as provided insights into acceptability and implementation. A realist review of preventative interventions, including qualitative and grey literature, may help to address these gaps.^50^

The COVID-19 pandemic has highlighted the persistence of health inequities in society and importance of addressing them in recovery plans. Although the evidence reviewed in this study is limited, it does suggest potential benefits of hospital-based preventative interventions for people experiencing homelessness. Policymakers and healthcare providers may consider implementing and evaluating person-centred multi-component preventative interventions. Our study has shown these types of interventions, which aim to provide an integrated and comprehensive approach to coordinating hospital and community-based services, plan for care and accommodation post-discharge, and provide advocacy, support, and assistance with social and welfare needs for people experiencing homelessness in hospital, are likely to be beneficial. Models of care using this type of approach already exist,^51^^,^^55^^,^^57^, ^58^, ^59^, ^60^, ^61^^,^^64^, ^65^, ^66^, ^67^^,^^69^^,^^71^^,^^73^ but have not been widely adopted in mainstream hospital care. An essential first step to any hospital-based preventative intervention is to take a more structured approach to identifying patients experiencing homelessness or at risk of becoming homeless, such as the use of ‘best practice alerts’ in electronic health records.^50^ Preventative interventions have been implemented in both inpatient and ED settings and may improve individual psychosocial outcomes, reduce unplanned healthcare utilisation and costs, and reduce health inequities amongst this marginalised population.

## Contributors

SL conceived and designed the study, reviewed the articles, extracted data, synthesised the results and drafted the manuscript. JD reviewed articles, checked data extraction, and reviewed the manuscript. RA, FS, ST, AH contributed to the design of the study and reviewed the manuscript. NH provided expert clinical advice to help develop the search strategy and reviewed the manuscript.

## Data sharing statement

All data extracted for this study are available upon request to the corresponding author.

## Declaration of interests

SL and JD are Fellows, NH is medical director, and AH is a trustee of the Pathway charity. Several studies of the Pathway model were reviewed in this paper. ST has received personal fees from Gilead Sciences and grants from the British HIV Association outside the submitted work.

## Appendix Supplementary materials

Image, application 1

Image, application 2

Image, application 3

Image, application 4
