# Supplementary material for: Hospital-based preventative interventions for people experiencing homelessness in high-income countries: A systematic review
Source: eClinicalMedicine. 2022 Oct 22;54:101657. doi: 10.1016/j.eclinm.2022.101657 (PMC9597099; doi:10.1016/j.eclinm.2022.101657)
Supplement: Supplementary file 1 [file mmc1.docx]

# Hospital-based preventative interventions for people experiencing homelessness in high-income countries: a systematic review

## Luchenski SA, Dawes J, Aldridge RW, Stevenson F, Tariq S, Hewett N, Hayward AC

### Appendix 1. List of Preventative Interventions for People Experiencing Homelessness.

Potential preventative intervention categories were identified from a scoping review of scientific and grey literature and expert opinion and mapped against version 10 of the International Classification of Disease (ICD-10) chapters. The purpose of the table was to inform the development of the intervention search terms for the systematic review. We have also indicated which ICD-10 chapters had papers identified in the systematic review or not to highlight research gaps.

| ****ICD-10 Chapter**** | ****Potential Preventative Interventions**** | ****Papers Identified in Systematic Review**** |
| --- | --- | --- |
| 1. Certain infectious and parasitic diseases | Vaccinations | Yes |
|  | Blood-Borne Viruses |  |
|  | Sexually Transmitted Infections |  |
|  | Tuberculosis |  |
| 1. Neoplasms | Breast, Cervical and Bowel Cancer | No |
| 1. Diseases of the blood and blood-forming organs and certain disorders involving the immune mechanism | None | No |
| 1. Endocrine, nutritional and metabolic diseases | Diabetes | No |
|  | Diet |  |
|  | Physical Activity |  |
| 1. Mental and behavioural disorders | Mental health and mental disorders | Yes |
|  | Alcohol |  |
|  | Drugs |  |
| 1. Diseases of the nervous system | Dementia, alcohol-related or otherwise or 'cognitive impairment' | No |
| 1. Diseases of the eye and adnexa | Sight | No |
| 1. Diseases of the ear and mastoid process | Hearing | No |
| 1. Diseases of the circulatory system | Cardiovascular disease | No |
| 1. Diseases of the respiratory system | Tobacco cessation | No |
| 1. Diseases of the digestive system | None | No |
| 1. Diseases of the skin and subcutaneous tissue | None | No |
| 1. Diseases of the musculoskeletal system and connective tissue | Falls and Musculoskeletal Health | No |
| 1. Diseases of the genitourinary system | None | No |
| 1. Pregnancy, childbirth and the puerperium | Contraception | No |
| 1. Certain conditions originating in the perinatal period | n/a | n/a |
| 1. Congenital malformations, deformations and chromosomal abnormalities | n/a | n/a |
| 1. Symptoms, signs and abnormal clinical and laboratory findings, not elsewhere classified | n/a | n/a |
| 1. Injury, poisoning and certain other consequences of external causes | Acquired Brain Injury/History of Brain Injury | No |
| 1. External causes of morbidity and mortality | Violence | No |
|  | Intentional Self-Harm |  |
| 1. Factors influencing health status and contact with health services | Integrated Care/Care Coordination/Case Management | Yes |
|  | Housing/Discharge Planning |  |
|  | Financial Support |  |
|  | Legal Support |  |
|  | Social Support |  |
|  | Support with GP registration/access to primary care and community services |  |
|  | Provision of Basic necessities |  |
|  | Intermediate care |  |
|  | Human trafficking and modern slavery |  |
|  | Other |  |
| 1. Codes for special purposes | n/a | n/a |
