# Supplementary material for: Hospital-based preventative interventions for people experiencing homelessness in high-income countries: A systematic review
Source: eClinicalMedicine. 2022 Oct 22;54:101657. doi: 10.1016/j.eclinm.2022.101657 (PMC9597099; doi:10.1016/j.eclinm.2022.101657)
Supplement: Supplementary file 2 [file mmc2.docx]

# Hospital-based preventative interventions for people experiencing homelessness in high-income countries: a systematic review

## Luchenski SA, Dawes J, Aldridge RW, Stevenson F, Tariq S, Hewett N, Hayward AC

### Appendix 2. Search Strategy

Use **Keywords** (consistent across all databases) and **Subject Headings** (specific to each database) to search in each database as follows:

(homeless keywords OR subject headings) AND (hospital keywords OR subject headings) AND (prevention interventions keywords OR subject headings)

**Keywords - Combine 1, 2, 3 with AND**

1. **Homelessness**
   - (homeless* OR 'no fixed address' OR 'no fixed abode' OR 'underhouse*' OR 'roofless*' OR 'seeking shelter' OR unhouse* OR 'sleeping rough' OR 'rough sleep*' OR 'unstabl* hous*' OR 'housing instability' OR 'precarious* hous*' OR hostel)
2. **Hospitals**
   - (hospital* OR 'secondary care' OR 'acute care' OR 'tertiary care' OR 'accident and emergency' OR 'emergency department' OR outpatient OR inpatient OR 'admitted patient' OR admission)
3. **Preventive Interventions**
   - (prevent* OR public health OR population health OR health promot* OR Social medicine OR vaccin* OR immun* OR screen* OR assess* OR diagnos* OR identif* OR test* OR refer* OR Early interven* OR Health education OR health information OR educat* OR inform* OR counsel* OR therap* OR brief advice OR brief intervention OR prophylaxis OR Surveillance OR registry OR Social determinant* OR wider determinants OR hous* OR financial support OR legal support OR reconnect* OR cloth* OR basic necessities OR basic needs OR toiletries OR food OR meal OR beverage OR Harm reduction OR needle exchange OR needle and syringe OR methadone OR opioid substitution therapy OR OST OR buprenorphine OR naloxone OR narcan OR drug treatment OR overdose OR alcohol treatment OR rehab* OR detox* OR Specialist care OR (fracture AND liaison) OR physiotherap* OR physical therap* OR podiatr* OR hearing test OR vision test OR dentistr* OR Contraception OR family planning OR long acting reversible contraception OR long-acting reversible contraception OR larc OR intrauterine device OR ((refer* OR regist*) AND (primary care OR general practice OR GP OR family doctor OR family physician)) OR (refer* AND (respite care OR medical respite OR intermediate care OR transitional care OR recovery centre)) OR Case manage* OR multidisciplinary team OR discharge plan* OR care plan OR pathway OR (peer OR social OR community OR friend OR buddy) AND (advoca* OR support OR help OR counsel*))

**Subject Headings:**

**Medline via Ovid (Combine 1, 2, 3 with AND)**

1. Exp Homeless persons/
2. Exp Hospitals OR Exp Hospital Medicine/ OR Exp Secondary Care/ OR Exp Tertiary Healthcare/ OR Exp Outpatients/ OR Exp Inpatients/ OR Exp Patient Admission/ OR Exp Hospitalization OR Exp Emergency Service, Hospital
3. Exp Preventive Health Services/ OR Exp Preventive Medicine/ OR Exp Public Health/ OR Exp Health Promotion/ OR Exp Social Medicine/ OR Exp Vaccination/ OR Exp Immunization/ OR Exp Mass Screening/ OR Exp Diagnosis/ OR Exp Referral and Consultation/ OR Exp Early Intervention / OR Exp Patient Education as Topic/ OR Exp Health Education/ OR Exp Counseling/ OR Exp Post-Exposure Prophylaxis OR Exp Pre-Exposure Prophylaxis/ OR Exp Dental Prophylaxis/ OR Exp Chemoprevention/ OR Exp Epidemiologic Methods/ OR Exp Social Determinants of Health/ OR Exp Sociological Factors/ OR Exp Housing/ OR Exp Financial Support/ OR Exp Clothing/ OR Exp Food/ OR Exp Beverage/ OR Exp Meals OR Exp Harm Reduction/ OR Exp Needle-Exchange Programs OR Exp Vision Tests/ OR Exp Hearing Tests/ OR Exp Podiatry/ OR Exp Preventive Dentistry/ OR Exp Public Health Dentistry/ OR Exp Physical Therapy Modalities/ OR Exp Contraception/ OR Exp Patient Care Planning/ OR Exp Patient Care Team/ OR Exp Continuity of Patient Care/ OR Exp Peer Group/ OR Exp Patient Advocacy/ OR Exp Social Environment/

**Embase Via Ovid (Combine 1, 2, 3 with AND)**

1. Exp Homelessness/
2. Exp Hospital OR Exp Hospital Medicine/ OR Exp Secondary Health Care/ OR Exp Tertiary Health Care/ OR Exp Outpatient/ OR Exp Hospital Patient/ OR Exp Hospital Admission/ OR Exp Hospitalization/ OR Exp Emergency Ward/
3. Exp Prevention/ OR Exp Preventive Health Service/ OR Exp Preventive Medicine/ OR Exp Public Health/ OR Exp Public Health Service/ OR Exp Health Promotion/ OR Exp Social Medicine/ OR Exp Vaccination/ OR Exp Immunization/ OR Exp Screening/ OR Exp Diagnosis/ OR Exp Patient Referral/ OR Exp Early Intervention / OR Exp Patient Education/ OR Exp Health Education/ OR Exp Counseling/ OR Exp Post Exposure Prophylaxis OR Exp Pre-Exposure Prophylaxis/ OR Exp Dental Prophylaxis/ OR Exp Chemoprophylaxis/ OR Exp Disease Surveillance/ OR Exp Register/ OR Exp Social Determinants of Health/ OR Exp Social Aspects and Related Phenomena/ OR Exp Housing/ OR Exp Financial Management/ OR Exp Clothing/ OR Exp Food/ OR Exp Beverage/ OR Exp Meal OR Exp Harm Reduction/ OR Exp Vision Test/ OR Exp Hearing Test/ OR Exp Podiatry/ OR Exp Preventive Dentistry/ OR Exp Physiotherapy/ OR Exp Contraception/ OR Long-Acting Reversible Contraception/ OR Exp Patient Care Planning/ OR Exp Patient Care/ OR Exp Peer Group/ OR Exp Patient Advocacy/

**PsychInfo Via Ovid**

1. Exp Homeless/
2. Exp Hospitals OR Exp Outpatients/ OR Exp Hospitalized Patients/ OR Exp Hospital Admission/ OR Exp Hospitalization OR Exp Emergency Services/
3. Exp Prevention/ OR Exp Preventive Medicine/ OR Exp Public Health/ OR Exp Public Health Services/ OR Exp Health Promotion/ OR Exp Immunization/ OR Exp Screening/ OR Exp Diagnosis/ OR Exp Professional Referral/ OR Exp Early Intervention / OR Exp Client Education/ OR Exp Health Education/ OR Exp Counseling/ OR Exp Social Issues/ OR Exp Housing/ OR Exp Clothing/ OR Exp Food/ OR Exp Harm Reduction/ OR Exp Dentistry/ OR Exp Physical therapy/ OR Exp Birth Control/ OR Exp Treatment Planning/ OR Exp Case Management/ OR Exp Peer Counseling/ OR Exp Advocacy/

**The Healthcare Management Information Consortium (HMIC) database via Ovid**

1. Exp Homelessness/
2. Exp Hospitals OR Exp Secondary Care/ OR Exp Tertiary Care/ OR Exp Out Patients/ OR Exp In Patients/ OR Exp Patient Admission/ OR Exp Hospitalisation/ OR Exp Hospital Patients/ OR Exp Accident & emergency services/
3. Exp Preventive Measures/ OR Exp Preventive Medicine/ OR Exp Public Health/ OR Exp Health Promotion/ OR Exp Social Medicine/ OR Immunisation/ OR Exp Screening/ OR Exp Diagnosis/ OR Exp Referral/ OR Exp Patient Education/ OR Exp Health Education/ OR Exp Counselling/ OR Exp Health Surveillance/ OR Exp Social Factors/ OR Exp Housing/ OR Exp Financial Support/ OR Exp Clothing/ OR Exp Food/ OR Exp Beverages/ OR Exp Meals/ OR Exp Harm Reduction/ OR Exp Drug Treatment Centres/ OR Exp Podiatry/ OR Exp Preventive Dentistry/ OR Exp Physiotherapy/ OR Exp Contraception/ OR Exp Health Care Teams/ OR Exp Continuity of Patient Care/ OR Exp Peer Groups/ OR Exp Patient Advocacy/ OR Exp Social Conditions/

**CINAHL Plus vis EbscoHost**

1. (MH "Homeless Persons") OR (MH "Homelessness")
2. (MH "Hospitals+") OR (MH “Hospital Medicine+”) OR (MH “Secondary Health Care+” OR (MH “Tertiary Health Care+” OR (MH “Inpatients+”) OR (MH “Patient Admission+”) OR (MH “Hospitalization+”) OR (MH “Emergency Service+”)
3. (MH “Preventive Health Care+” OR (MH “Public Health+”) OR (MH “Health Promotion+”) OR (MH “Health Screening+”) OR (MH “Diagnosis+”) OR (MH “Referral and Consultation+”) OR (MH “Early Intervention+”) / OR (MH “Patient Education+”) OR (MH “Health Education+”) OR (MH “Counseling+”) OR (MH “Dental Prophylaxis+”) OR (MH “Chemoprevention+”) OR (MH “Social Determinants of Health+”) OR (MH “Social Problems+”) OR (MH “Housing+”) OR (MH “Financial Support+”) OR (MH “Clothing+”) OR (MH “Food+”) OR (MH “Beverages+”) OR (MH “Meals+”) OR (MH “Harm Reduction+”) OR (MH “Needle Exchange Programs+”) OR (MH “Podiatry+”) OR (MH “Preventive Dentistry+”) OR (MH “Public Health Dentistry+”) OR (MH “Physical Therapy+”) OR (MH “Contraception+”) OR (MH “Patient Care Plans+”) OR (MH “Multidisciplinary Patient Care Team+”) OR (MH “Continuity of Patient Care+”) OR (MH “Case Management+”) OR (MH “Peer Group+”) OR (MH “Patient Advocacy+”) OR (MH “Support, Psychosocial+”)

**Web of Science Core Collection (Note: TS = Topic) - 2047**

1. (TS = (homeless* OR no fixed address' OR 'no fixed abode' OR 'underhouse*' OR 'roofless*' OR 'seeking shelter' OR unhouse* OR 'sleeping rough' OR 'rough sleep*' OR 'unstabl* hous*' OR 'housing instability' OR 'precarious* hous*' OR hostel)) AND LANGUAGE: (English) - 19,534
2. (TS = (hospital* OR 'secondary care' OR 'acute care' OR 'tertiary care' OR 'accident and emergency' OR 'emergency department' OR outpatient OR inpatient OR 'admitted patient' OR admission)) AND LANGUAGE: (English) - 1,159,781
3. TS = (prevent* OR public health OR population health OR health promot* OR Social medicine OR vaccin* OR immun* OR screen* OR assess* OR diagnos* OR identif* OR test* OR refer* OR Early interven* OR Health education OR health information OR educat* OR inform* OR counsel* OR therap* OR brief advice OR brief intervention OR prophylaxis OR Surveillance OR registry OR Social determinant* OR wider determinants OR hous* OR financial support OR legal support OR reconnect* OR cloth* OR basic necessities OR basic needs OR toiletries OR food OR meal OR beverage OR Harm reduction OR needle exchange OR needle and syringe OR methadone OR opioid substitution therapy OR OST OR buprenorphine OR naloxone OR narcan OR drug treatment OR overdose OR alcohol treatment OR rehab* OR detox* OR Specialist care OR (fracture AND liaison) OR physiotherap* OR physical therap* OR podiatr* OR hearing test OR vision test OR dentistr* OR Contraception OR family planning OR long acting reversible contraception OR long-acting reversible contraception OR larc OR intrauterine device OR ((refer* OR regist*) AND (primary care OR general practice OR GP OR family doctor OR family physician)) OR (refer* AND (respite care OR medical respite OR intermediate care OR transitional care OR recovery centre)) OR Case manage* OR multidisciplinary team OR discharge plan* OR care plan OR pathway OR (peer OR social OR community OR friend OR buddy) AND (advoca* OR support OR help OR counsel*)) AND LANGUAGE: (English) - 17, 522, 885

**Cochrane Library (Search ‘Title, Abstract, Keyword’, limit to 01/01/1999 - ) - 43 Cochrane Reviews, 353 Trials**

1. Homeless Keywords as above
2. Hospital Keywords as above
3. Don’t search interventions
