# Supplementary material for: Hospital-based preventative interventions for people experiencing homelessness in high-income countries: A systematic review
Source: eClinicalMedicine. 2022 Oct 22;54:101657. doi: 10.1016/j.eclinm.2022.101657 (PMC9597099; doi:10.1016/j.eclinm.2022.101657)
Supplement: Supplementary file 3 [file mmc3.docx]

# Hospital-based preventative interventions for people experiencing homelessness in high-income countries: a systematic review

## Luchenski SA, Dawes J, Aldridge RW, Stevenson F, Tariq S, Hewett N, Hayward AC

### Appendix 3. Results from Individual Included Studies.

***Note:** The Pathway model is reported in several different studies with differing descriptions of the intervention because there is variability in how the model is implemented in different hospitals and how the model is reported. We produced Pathway intervention descriptions based on how they were reported in the individual studies.

| Reference | Setting | Study Population | Sample Size  (% PEH) | Intervention | Comparison | Outcome | Quality |
| --- | --- | --- | --- | --- | --- | --- | --- |
| Abdel-Baki (2018)^79^ | Two academic hospitals with specialist mental health services in Montreal, Canada. | First-episode psychosis inpatients with co-morbid SUD. | 237  (21.9%) | Long-acting injectable antipsychotics given as first line medication to those with first episode psychosis and concurrent substance use disorder | Those who received oral antipsychotics first. | Significantly longer mean time to first psychotic relapse; no difference in rehospitalisation rates | Weak |
| Albanese (2016)^67^ | 52 hospitals in England where a 'Homeless Hospital Discharge Fund' pilot project was in place, UK | PEH admitted to hospital. | Unknown (100%) | Six homeless hospital discharge interventions, each with a different team/approach: 1) housing link workers; 2) nursing link workers; 3) nursing and housing link workers; 4) *Pathway teams (GP-led care coordination); 5) project workers (one of the above) plus accommodation; 6) accommodation only | None | Homeless hospital discharge projects appear to improve accommodation availability at discharge | Weak |
| Bernstein 2007^82^ | 14 academic EDs across the USA. | Adults who screened positive for drinking over the National Institute of Alcohol Abuse and Alcoholism low-risk limits. | 1132 (11%) | Screening for high risk alcohol drinking, brief intervention (Brief Negotiated Interview) to reduce unhealthy alcohol use, and a written handout of local referral resources. | Screening for high risk alcohol drinking and written handout of local referral resources. | Drinking behaviour in the past 30 days (quantity, frequency, and maximum use) | Moderate |
| Castillo (2018)^55^ | Single academic medical center ED in the downtown area of San Diego, USA where a large proportion of the region’s homeless population live. | Patients identified as homeless in the hospital electronic health record. | 1129  (100%) | Automated computerised alert (Best Practices Alert) embedded within the hospital electronic health record to prompt clinicians to order and administer a hepatitis A vaccine for PEH during an outbreak. | 6-month historical control prior to outbreak, 6-month control during outbreak but prior to intervention, and 6-month intervention period | EHR alert systems highly beneficial for prompting clinicians to administer vaccines to homeless populations during a community outbreak of Hep A | Moderate |
| Cummings (2006)^80^ | ED of an inner-city hospital in Edmonton, Canada which serves ‘high risk’ populations, including PEH, people who use substances, working poor and/or recent immigrants. | ED patients | 2366  (unknown) | Comprehensive health needs assessment using standardised instruments and nurse-led interview. Health promotion and disease prevention interventions that could be delivered during ED waiting time were offered and/or participants referred to community or inpatient services. Interventions included screening and counselling for smoking, alcohol and drug use, immunisations, and Pap smears. | Before interventions and at 30, 60, and 90 days after the initial ED visit | Only a small proportion of patients who attended the ED (<5%) benefited from the interventions, but the programme did not seem to negatively affect ED workflow or waiting times. | Weak |
| Evans (2018)^68^ | Central London academic hospital ED. | All ED patients aged 16yrs+ having routine blood tests taken during a 6-week period. Patients were classified according to a binary residency variable (fixed abode/no fixed abode). | 13,179 (>2%) | Opt-out screening and linkage to care for hepatitis B and C. Treatment offered was according to UK national clinical guidelines. | No comparison group | Opt-out HCV/HBV screening is acceptable to PEH in the ED. However, PEH and people who use drugs were more difficult to contact about their results and get linked into care than other groups. | Weak |
| Field (2019)^69^ | Hospitals in seven locations across England (Bradford, Brighton, Manchester and four London-based teams) who had a ‘Pathway’ team. | All patients experiencing homelessness who were referred to the Pathway team. | 1009  (100%) | Pathway* model: GP-led specialist in-reach hospital care coordination. Teams provide advocacy, advice, and support for both the patient and the admitting clinical team and liaise with community health and housing services for care post-discharge. | 120 days before index admission and 120 days after discharge from index admission | Unplanned admissions and A&E attendances were not reduced, but planned hospital care increased which is positive as it suggests patients are receiving necessary follow-up care. Most patients maintained or improved their housing status on discharge. | Moderate |
| Forchuk (2008)^81^ | Acute care psychiatric ward within a general hospital and a tertiary care psychiatric hospital in London, Ontario, Canada | In-patients with diagnosis of serious mental illness at risk of being discharged to 'no fixed address'. | 14  (100%) | A housing advocate provided assistance finding housing to prevent homelessness post hospital discharge plus fast-tracked income support to cover first and last month’s rent. | Usual care - no direct and immediate assistance with housing but included referral to social work for housing support if requested by the healthcare team. | All individuals in the intervention group obtained and maintained housing vs none in the control group. | Moderate |
| Gabrielian (2017)^56^ | ED of Veterans Affairs Greater Los Angeles, USA. The catchment area had 6371 homeless veterans on any given night. | Veterans who were identified as homeless or at risk of homeless following formal homelessness screening by the ED triage nurses. | 112  (100%) | Homelessness screening by ED triage nurses using a 3-item instrument. Patients who screened positive offered choice of an ED visit or a specialist homeless primary care visit co-located within the ED. Primary care team was interdisciplinary and had prior experience working with PEH (primary care physician, mental health clinical nurse specialist, and clerks who worked with homeless/mental health patients). Staff tracked referrals and social service needs and offered patients longitudinal follow-up. | No comparison group | It is feasible to co-locate a primary care clinic within an ED and recruit homeless patients to attend. Acceptability from the perspective of ED clinicians was mixed. | Weak |
| Grover (2018)^57^ | ED of a suburban community hospital in Monterey, California, USA | Frequent users of the ED | 158  (7.6%) | Emergency Department Recurrent Visitor Program (EDRVP): multidisciplinary case management/care planning delivered in the ED by an ED social worker or registered nurse (RN), with emergency physicians, social workers, ED RNs, chemical dependency providers, behavioural health RNs, case managers, and representatives from local insurance providers. | 1 year before and 1 year after enrolment into the programme | ED visits, inpatient admissions, imaging services, length of stay, and costs were all reduced by the intervention. | Moderate |
| Hewett (2016)^70^ | Two large inner-city hospitals in London and Brighton, UK. | Homeless adult inpatients. | 410  (100%) | Pathway* model: multidisciplinary care coordination, advocacy, and hospital discharge planning. A specialist nurse provided support and established community links. A GP performed ward rounds and provided advocacy, advice and medical input. Multiagency meetings involved Pathway team, local council officers, hostel managers, outreach workers, drug and alcohol nurses, homeless centre staff, social and palliative care workers, hospital consultants and therapists. | Standard care: patients visited by nurse once per week and provided with a leaflet about service. They were not discussed in multi-agency care planning meetings. | Quality of life improved, but it was not statistically significant. The proportion of people sleeping on the streets reduced more in the intervention group than standard care. There was no effect on length of stay, ED re-attendances, or re-admissions. The intervention was shown to be cost-effective using UK national guidelines. | Strong |
| Hutton (2019)^75^ | ED of an inner-city tertiary referral hospital in Melbourne, Australia. | All adult ED patients. | 1122  (2%) | Hepatitis C (HCV) risk factor screening questionnaire to determine who to offer testing to; point-of-care oral anti-HCV test to those who screened positive for a risk factor; HCV serology testing for those whose point-of care test was positive; offer of direct acting antiviral treatment in clinic for those with a confirmatory serology test. | None | Among those with a confirmed serology test, uptake and continuation of HCV treatment was low overall, and particularly poor for PEH. Among those who were engaged in treatment, all housed patients achieved a cure and none of the PEH were cured. | Weak |
| James (2009)^58^ | ED of the Boston Medical Center, an inner-city hospital which is the largest provider of care for deprived patients in Massachusetts, USA | Patients over 21 years who were in a high-risk category: homeless, recent history of substance abuse, or recent or current incarceration. | 122  (74%) | Hepatitis A vaccination offered to patients in the ED during a city-wide outbreak/ vaccination campaign. | None | The study demonstrated that it is feasible to rapidly give vaccinations in the ED to PEH to stem an epidemic. | Weak |
| Kang (2020)^59^ | ED and inpatient care at an inner-city academic hospital in San Diego, California, USA. The hospital was located at the epicenter of the acute hepatitis A outbreak which is addressed in this study. | Self-identified PEH seeking care in the ED or admitted to hospital for hepatitis A. | 1374 (100%) | Hospital-level hepatitis A prevention and outbreak management strategy: 1) vaccinate (flags in electronic health records of self-identified PEH); 2) sanitise (enhanced measures for the hospital and personal hygiene kits provided to PEH and at-risk groups); and 3) educate (information distributed by nurses with hygiene kits and email newsletters to general public). Initially PEH were kept in hospital until they were no longer infectious and later on they were offered housing so they could be discharged if they were otherwise well. | None | Vaccinations helped to slow and eventually end the outbreak. Introduction of housing for potentially infectious homeless patients reduced length of stay in hospital. | Weak |
| Khan (2019)^71^ | Large inner-city specialist mental health hospital in London, UK. | Patients admitted to the mental health trust who were homeless or vulnerably housed and without a care coordinator, irrespective of their right to statutory entitlements, nationality, or local area connection. | 237  (100%) | Pathway* model: GP-led specialist homeless team with a mental health practitioner, housing worker, and business manager. Roles: specialist clinical review, advice, and advocacy; discharge planning; multidisciplinary care planning; support with housing and welfare benefits applications and GP registration; access to legal advice and necessities such as mobile phone, foodbank vouchers and subsistence; specialist expertise in patients with no recourse to public funds, mental capacity act, mental health act, safeguarding, modern day slavery and trafficking. | Baseline and after discharge. | Housing status improved after discharge. | Weak |
| Khan (2020)^72^ | Large inner-city specialist mental health hospital in London, UK. | Patients referred from mental health inpatient wards who were homeless or vulnerably housed and not currently linked to a Community Mental Health Team. | 61  (100%) | Pathway* Model: same as Khan 2019 above | Baseline, 3 months, and 6 months after intervention | Total mean costs were reduced at 3 months. Follow-up rate was too low at 6 months to draw conclusions. | Weak |
| Killaspy (2004)^73^ | Inner-city mental health and social care hospital in central London, UK. | All clients of a community mental health outreach team for PEH admitted to a mental health inpatient facility. | 50  (100%) | Specific 12-bed inpatient facility for homeless clients of the Focus Outreach team. Hypothesis is that clients admitted to this facility would have better discharge planning than standard care. | Standard care: clients who were eligible for the intervention, but were unable to receive because of space limitations were hospitalised in different facilitites. | Both groups were equally likely to be discharged to stable accommodation and to be residing in stable accommodation at 12 months. Engagement and medication compliance improved in intervention vs control. | Weak |
| Lintzeris (2020)^76^ | EDs of two hospitals in Southeastern Sydney Local Health District, Australia | Patients >18 years with moderate to severe substance used disorder, ED attendance on at least five occasions in the past year, not already engaged in another care co-ordination programme | 46  (23%) | Integrated Management Pathways for Alcohol and drug Clients into Treatment (IMPACT) Service: assertive case management programme for ~6 months with 2 full-time social workers and 0.1 FTE of an Addiction Medicine Specialist. Services included: multidisciplinary needs assessment; multiagency care planning; supported referrals to relevant health and welfare services; hospital ED management plan with alerts on the client’s electronic health records; transport assistance and brokerage funding to assist with essential one-off expenses. Upon completion, care transferred to an agreed community provider. | Pre/post study of healthcare use and costs in intervention clients vs clients assessed as eligible but who did not engage. Clinical/welfare outcomes measured before/after in intervention group only. | Greater reductions in ED presentations, hospital admissions and costs were seen in the intervention vs control . The intervention group also reduced their substance use significantly. There were no significant differences in health outcomes, housing status, violence/arrest, or employment/education before/after the intervention. | Weak |
| McCormack (2013)^60^ | ED of an academic, urban, public hospital in New York City, USA | Chronically homeless, alcohol-dependent adults with persistently frequent ED use. | 60  (100%) | Case management and homeless outreach: social worker and outreach team met with participants, guided by previously developed care plans to offer shelter on discharge. Assigned case-workers relocated participants into increasingly supportive settings, coordinated multidisciplinary care, and updated plans on the basis of participants’ medical, psychosocial, and housing needs. | 6m before/after the intervention with prospective controls (same patient pool as intervention, but received standard care) and retrospective controls (from the previous year using the same algorithm for patient identification and who were alive for the entire observation period) | The intervention reduced ED visits and improved housing for intervention participants vs controls. Findings for reductions in inpatient visits were less clear, but also indicated a trend toward greater reductions for the intervention group. | Moderate |
| Merchant (2018)^61^ | Two urban EDs in Providence, Rhode Island, USA | Adult ED patients needing a drug use intervention | 1030 (20.1%) | Screening for problem drug use, 20–30min brief intervention to motivate participants to reduce their drug misuse and seek appropriate treatment. The BI sessions were led by a research assistant and based on motivational interviewing and the health beliefs model. BI arm participants were contacted via telephone for a booster session by the same RA 2–4weeks post-ED enrolment. | Control group were screened and completed study questionnaires only | The intervention did not decrease drug use or increase drug treatment services utilisation more than the control. | Moderate |
| Nossel (2016)^62^ | Psychiatric ED of a large academic urban hospital in New York City, USA | Frequent users (three or more visits in the previous year) of EDs for psychiatric reasons | 97  (63%) | Project Connect: time limited (up to 6 months) peer-led "critical time" care coordination to help vulnerable individuals transition from hospital to community settings. Team comprised of three full-time peer critical time intervention specialists, a half-time clinical director, and a psychiatrist (.1 FTE). Peer specialists had experienced mental illness, substance abuse, or homelessness themselves. Peers advocated for and supported clients with high-quality discharge plans, psychiatric treatment, substance use treatment, access to medical care, housing, benefits, family involvement, self-management skills, return to work or school, and enhancing hope. | Standard care: eligible for intervention but not referred because of limited capacity. | A&E and inpatient use (combined) reduced in both groups. Outpatient use increased in intervention group and decreased in control group. | Moderate |
| Okin (2000)^63^ | ED of an urban general hospital in San Francisco, California USA | ED frequent users | 53  (67%) | Comprehensive, intensive case management model led by a master’s level psychiatric social worker. Case manager provided and coordinated all needed services: crisis intervention, individual and group supportive therapy, arrangement of stable housing and financial entitlements, harm reduction services and referral to substance use treatment, liaison with other community agencies and extensive, persistent outreach. | 12 months before and after intervention; no control group. | Number of ED visits decreased, number of medical outpatient visits increased, no changes in medical inpatient admissions or bed days or psychiatric inpatient admissions, bed days or psychiatric emergency visits. Hospital service costs were lower after intervention. Homelessness decreased, problem alcohol decreased, problem drug use decreased, percentage of patients without medicaid decreased, number of patients without a primary care provider decreased. | Weak |
| Phillips (2006)^77^ | Inner metropolitan tertiary hospital in Melbourne, Australia | Adult frequent attenders (more than 6 times per year) of the ED with case management initiated | 60  (43%) | Highly flexible, multidisciplinary case management that was responsive to the needs of the patient. The model combined hospital-based care, community and primary health care, and short- and long-term case management. | 12 months before and after intervention; no control group. | No significant changes in ED attendance, length of stay, or admissions. Housing status increased, primary care linkage and community care engagement increased, drug and alcohol use were unaffected. | Weak |
| Raven (2011)^64^ | A large urban safety net public hospital for a diverse and primarily underserved population in New York City, USA | Adult patients who were at 'high risk' for future hospital admissions (as determined by an algorithm). | 19  (89%) | Patient-centred, intensive, and flexible case management tailored to the individual. Intervention began at patients’ bedside during hospital admission and extended into community after discharge. Case managers helped with things such as: housing, primary care access, transportation to and advocacy during appointments, medication management, entitlements enrolment, improved connections to psychiatric and substance use treatment, home visits and necessities such as mobile phones. | 12 months before and after intervention; no control group. | Inpatient and ED visits decreased; outpatient visits increased; and medicaid costs reduced before and after the intervention. | Moderate |
| Sadowski (2009)^65^ | A public teaching hospital and a private, non-profit hospital in Chicago, Illinois, USA | Social worker referred homeless adult inpatients with chronic illnesses | 405  (100%) | Intervention had 3 integrated components: provision of transitional housing at respite care centres, subsequent placement in stable housing, and multidisciplinary case management. Case management led by an intervention master’s level social worker on-site at the hospitals, the respite care facilities and stable housing sites. | Usual care - referral to usual hospital social worker and received usual discharge planning services with no continued relationship after hospital discharge. | Reduction in number of admissions, hospital bed days and ED visits in intervention compared to control. | Strong |
| Shumway (2008)^66^ | An urban public general hospital in San Francisco, USA | ED frequent users with psychosocial problems that could be addressed with case management | 252  (81%) | Long-term clinical case management that included assessment, crisis intervention, individual and group supportive therapy, assistance in obtaining stable housing and income entitlements, linkage to medical care providers, referral to substance use services when needed, ongoing assertive community outreach to maintain continuity of care. Master's-level psychiatric social workers provided most case management services in collaboration with a nurse practitioner, a primary care physician, and a psychiatrist. | Usual care. Patients were eligible to receive case management services at the conclusion of the 24 month study. | Compared to the control group, the intervention improved homelessness, problem alcohol use, health insurance, basic financial needs, ED visits, medical inpatient admissions, ED costs. There was no difference in psychiatric symptoms (Total Brief Symptom Inventory (BSI) Score), medical inpatient days, psychiatric emergency visits, psychiatric inpatient admissions, psychiatric inpatient days, medical outpatient visits, total hospital service costs, costs for inpatient, psychiatric and outpatient services. | Moderate |
| Wood (2019)^78^ | An inner-city hospital which serves a large proportion of PEH in Perth, Australia | Homeless inpatients who were assessed as 'highly vulnerable'. | 44  (100%) | Integrated health and Housing First collaboration to re-house highly vulnerable patients and provide ongoing continuity of care. The homeless hospital team (GP-in-reach team based on the UK Pathway* model) identified vulnerable in-patients and referred them to Housing First programme for Perth's most chronic and complex rough sleepers. A specialist homeless multi-site GP practice provided continuity of care after discharge from hospital. | 12 months pre- and post-the date clients were housed for those who were housed for 12 months or more | Number of hospital admissions decreased, inpatient length of stay decreased, ED presentations reduced, ED re-presentations within 7 and 30 days reduced, intervention was cost saving (i.e. costs reduced) | Weak |
| Wyatt (2017)^74^ | Large, inner-city academic urban public hospital in London, UK | Homeless inpatients referred to the Pathway team | 396  (100%) | Pathway* model: multidisciplinary care coordination, GP and specialist nurse-led ward rounds, patient advocacy in relation to discharge and outpatient arrangements, and liaison with housing, legal and community medical representatives. Peer care navigators provide support and understanding and follow-up if necessary. | 90 days before and after intervention, no control group | Reduction in ED attendance, hospital admission, and length of stay | Weak |
