# Supplementary material for: Hospital-based preventative interventions for people experiencing homelessness in high-income countries: A systematic review
Source: eClinicalMedicine. 2022 Oct 22;54:101657. doi: 10.1016/j.eclinm.2022.101657 (PMC9597099; doi:10.1016/j.eclinm.2022.101657)
Supplement: Supplementary file 4 [file mmc4.docx]

Hospital-based preventative interventions for people experiencing homelessness in high-income countries: a systematic review

Luchenski SA, Dawes J, Aldridge RW, Stevenson F, Tariq S, Hewett N, Hayward AC

**Appendix 4. Quality Rating of Included Studies**

The quality assessment tool is available here <https://www.ephpp.ca/PDF/Quality%20Assessment%20Tool_2010_2.pdf>

The quality assessment dictionary is available here <https://www.ephpp.ca/PDF/QADictionary_dec2009.pdf>

|  | **Selection Bias** | | | **Study Design** | | | | | **Confounders** | | | **Blinding** | | | **Data Collection** | | | **Withdrawals and Dropouts** | | | **Intervention Integrity** | | | **Analyses** | | | | **Overall Rating** |
| --- | --- | --- | --- | --- | --- | --- | --- | --- | --- | --- | --- | --- | --- | --- | --- | --- | --- | --- | --- | --- | --- | --- | --- | --- | --- | --- | --- | --- |
| **Reference** | **Q1** | **Q2** | **Score** | **Q1** | **Q2** | **Q3** | **Q4** | **Score** | **Q1** | **Q2** | **Score** | **Q1** | **Q2** | **Score** | **Q1** | **Q2** | **Score** | **Q1** | **Q2** | **Score** | **Q1** | **Q2** | **Q3** | **Q1** | **Q2** | **Q3** |  |  |
| Abdel-Baki (2018)^79^ | 2 | 5 | 2 | Cohort analytic | N | n/a | n/a | 2 | 1 | 4 | 3 | 3 | 3 | 3 | 1 | 1 | 1 | 1 | 1 | 1 | 3 | 1 | 4 | I | I | 1 | 1 | Weak |
| Albanese (2016)^67^ | 4 | 5 | 3 | Mixed methods evaluation | N | n/a | n/a | 3 | 3 | 4 | 3 | 3 | 3 | 3 | 2 | 2 | 3 | 4 | 5 | n/a | 4 | 3 | 6 | O | O | 3 | 3 | Weak |
| Bernstein 2007^82^ | 2 | 3 | 3 | Cohort analytic | N | n/a | n/a | 2 | 1 | 1 | 1 | 3 | 3 | 2 | 1 | 1 | 1 | 1 | 2 | 2 | 1 | 3 | 5 | I | I | 1 | 1 | Moderate |
| Castillo (2018)^55^ | 2 | 1 | 2 | Cohort one group pre/post | N | n/a | n/a | 2 | 2 | 1 | 1 | 3 | 3 | 2 | 3 | 3 | 3 | 4 | 4 | n/a | 2 | 1 | 5 | I | I | 1 | 1 | Moderate |
| Cummings (2006)^80^ | 3 | 3 | 3 | Cohort one group pre/post | N | n/a | n/a | 2 | 3 | 4 | 3 | 3 | 3 | 2 | 1 | 1 | 1 | 1 | 3 | 3 | 3 | 2 | 2 | I | I | 1 | 2 | Weak |
| Evans (2018)^68^ | 4 | 3 | 3 | Cross-sectional | N | n/a | n/a | 3 | 1 | 2 | 2 | 3 | 3 | 2 | 2 | 2 | 2 | 1 | 2 | 2 | 3 | 2 | 5 | I | I | 1 | 1 | Weak |
| Field (2019)^69^ | 1 | 1 | 1 | Cohort one group pre/post | N | n/a | n/a | 2 | 2 | 1 | 1 | 1 | 2 | 2 | 2 | 2 | 3 | 1 | 1 | 1 | 1 | 2 | 6 | I | I | 1 | 2 | Moderate |
| Forchu~~c~~k (2008)^81^ | 4 | 5 | 3 | RCT | Y | Y | Y | 1 | 2 | 1 | 1 | 3 | 3 | 2 | 1 | 1 | 1 | 1 | 1 | 1 | 1 | 1 | 5 | I | I | 1 | 1 | Moderate |
| Gabrielian (2017)^56^ | 4 | 2 | 3 | Cross-sectional | N | n/a | n/a | 3 | 3 | 4 | 3 | 3 | 3 | 2 | 1 | 1 | 1 | 4 | 4 | 2 | 2 | 2 | 5 | I | I | 1 | 3 | Weak |
| Grover (2018)^57^ | 1 | 1 | 1 | Cohort (one group pre and post) | N | n/a | n/a | 2 | 2 | 1 | 1 | 1 | 2 | 2 | 3 | 3 | 3 | 4 | 2 | 1 | 1 | 3 | 6 | I | I | 1 | 1 | Moderate |
| Hewett (2016)^70^ | 2 | 2 | 2 | RCT | Y | Y | Y | 1 | 2 | 1 | 1 | 1 | 2 | 2 | 1 | 1 | 1 | 1 | 1 | 1 | 4 | 2 | 5 | I | I | 1 | 1 | Strong |
| Hutton (2019)^75^ | 2 | 2 | 2 | Interrupted Time Series | N | N | N | 2 | n/a | n/a | 2 | 1 | 1 | 3 | 2 | 2 | 3 | 1 | 3 | 3 | 3 | 2 | 6 | I | I | 1 | 1 | Weak |
| James (2009)^58^ | 3 | 5 | 3 | Interrupted Time Series | N | N | N | 3 | 3 | 4 | 3 | 1 | 3 | 3 | 2 | 2 | 3 | 2 | 4 | 3 | 4 | 2 | 4 | I | I | 2 | 2 | Weak |
| Kang (2020)^59^ | 3 | 4 | 3 | Interrupted Time Series | N | N | N | 2 | 3 | 3 | 3 | 1 | 2 | 2 | 1 | 1 | 1 | 2 | 4 | 3 | 4 | 2 | 4 | I | I | 1 | 1 | Weak |
| Khan (2019)^71^ | 3 | 3 | 3 | Cross-sectional | N | N | N | 3 | 3 | 3 | 3 | 1 | 3 | 3 | 2 | 2 | 3 | 4 | 5 | n/a | 4 | 2 | 6 | I | I | 3 | 3 | Weak |
| Khan (2020)^72^ | 3 | 5 | 3 | Cohort - pre/post | N | N | N | 2 | 3 | 3 | 3 | 1 | 3 | 3 | 1 | 1 | 1 | 1 | 3 | 3 | 4 | 2 | 6 | I | I | 1 | 2 | Weak |
| Killaspy (2004)^73^ | 3 | 2 | 3 | Cohort analytic (two group pre + post) | N | N | N | 2 | 1 | 2 | 2 | 1 | 1 | 3 | 1 | 1 | 1 | 1 | 2 | 2 | 1 | 2 | 6 | I | I | 1 | 2 | Weak |
| Lintzeris (2020)^76^ | 3 | 1 | 3 | Cohort analytic (two group pre + post) | N | N | N | 2 | 1 | 1 | 2 | 2 | 3 | 3 | 1 | 2 | 2 | 1 | 2 | 2 | 2 | 1 | 6 | I | I | 1 | 1 | Weak |
| McCormack (2013)^60^ | 3 | 5 | 3 | Controlled clinical trial | N | N | N | 1 | 2 | 1 | 1 | 3 | 3 | 2 | 1 | 1 | 1 | 1 | 1 | 1 | 1 | 2 | 5 | I | I | 1 | 1 | Moderate |
| Merchant (2018)^61^ | 2 | 5 | 2 | Randomised controlled trial | Y | Y | Y | 1 | 1 | 1 | 1 | 1 | 2 | 2 | 1 | 1 | 1 | 1 | 3 | 3 | 1 | 1 | 6 | I | I | 1 | 1 | Moderate |
| Nossel (2016)^62^ | 2 | 2 | 2 | Cohort analytic (two group pre+post) | N | N | N | 2 | 1 | 3 | 3 | 3 | 3 | 2 | 1 | 1 | 1 | 4 | 4 | 2 | 4 | 2 | 6 | I | I | 2 | 1 | Moderate |
| Okin (2000)^63^ | 3 | 3 | 3 | Cohort (pre/post) | N | N | N | 2 | 2 | 1 | 1 | 3 | 3 | 2 | 2 | 2 | 1 | 2 | 4 | 3 | 1 | 2 | 6 | I | I | 1 | 1 | Weak |
| Phillips (2006)^77^ | 3 | 3 | 3 | Cohort (pre/post) | N | N | N | 2 | 2 | 1 | 2 | 2 | 3 | 2 | 3 | 1 | 3 | 2 | 4 | 3 | 1 | 3 | 6 | I | I | 1 | 1 | Weak |
| Raven (2011)^64^ | 2 | 2 | 2 | Cohort (pre/post) | N | N | N | 2 | 2 | 4 | 1 | 3 | 3 | 2 | 1 | 1 | 1 | 1 | 3 | 3 | 3 | 2 | 2 | I | I | 1 | 1 | Moderate |
| Sadowski (2009)^65^ | 2 | 1 | 2 | RCT | Y | Y | Y | 1 | 2 | 1 | 1 | 1 | 1 | 1 | 1 | 1 | 1 | 1 | 2 | 2 | 1 | 2 | 5 | I | I | 1 | 1 | Strong |
| Shumway (2008)^66^ | 2 | 1 | 2 | RCT | Y | N | can't tell | 1 | 1 | 1 | 1 | 3 | 3 | 2 | 2 | 2 | 3 | 1 | 1 | 1 | 4 | 2 | 6 | I | I | 1 | 1 | Moderate |
| Wood (2019)^78^ | 5 | 5 | 3 | Cohort (one group pre/post) | N | n/a | n/a | 2 | 3 | 4 | 3 | 2 | 2 | 2 | 1 | 1 | 1 | 4 | 4 | 2 | 1 | 1 | 5 | I | I | 1 | 1 | Weak |
| Wyatt (2017)^74^ | 4 | 5 | 3 | Cohort (one group pre/post) | N | n/a | n/a | 2 | 3 | 4 | 3 | 2 | 2 | 2 | 1 | 1 | 1 | 4 | 5 | 2 | 4 | 2 | 6 | I | I | 3 | 3 | Weak |
